# Laparoscopic distal pancreatectomy for intraductal papillary mucinous neoplasm-associated pancreatic cancer: A case report

**DOI:** 10.1016/j.ijscr.2021.106376

**Published:** 2021-09-10

**Authors:** Hiroki Kaida, Yoshihiro Miyasaka, Marina Morishita, Nobuhiko Koreeda, Yousuke Hirano, Toshihiro Ohmiya, Shugo Uwatoko, Makoto Kawamoto, Akira Komono, Ryohei Sakamoto, Ryosuke Shibata, Daijiro Higashi, Satoshi Nimura, Masato Watanabe

**Affiliations:** a Department of Surgery, Fukuoka University Chikushi Hospital, 1-1-1 Zokumyoin, Chikushinoshi, Fukuoka 818-8502, Japan; b Department of Pathology, Fukuoka University Chikushi Hospital, 1-1-1 Zokumyoin, Chikushinoshi, Fukuoka 818-8502, Japan

**Keywords:** IPMN, Pancreatic cancer, Laparoscopic pancreatectomy, Esophagogastric junction cancer, Minimally invasive esophagectomy

## Abstract

**Introduction and importance:**

Intraductal papillary mucinous neoplasm (IPMN) of the pancreas is often found incidentally during examination for other diseases. In addition to the risk of malignant transformation, patients with IPMN are at risk of developing pancreatic cancer. We report a case of pancreatic tail cancer that developed separately from a preexisting IPMN after minimally invasive esophagectomy for cancer of the esophagogastric junction and was resected successfully by laparoscopic distal pancreatectomy.

**Case presentation:**

A 72-year-old man underwent thoracoscopic and laparoscopic esophagectomy for esophagogastric junction cancer. He had undergone surgery for ascending colon cancer 20 years ago. At that time, IPMN was confirmed in the pancreatic body by a preoperative examination. Computed tomography was regularly performed for postoperative work-up and follow-up of the IPMN, and a solid lesion with cystic components was detected in the pancreatic tail 9 months after the operation. On detailed examination, pancreatic ductal adenocarcinoma concomitant with IPMN, accompanied by a retention cyst, was considered. Laparoscopic distal pancreatectomy was successfully performed after neoadjuvant chemotherapy. Pathological diagnosis of the lesion in the pancreatic tail was of an invasive intraductal papillary mucinous carcinoma (ypT3ypN0yM0 ypStageIIA).

**Clinical discussion:**

If an IPMN is detected during preoperative examination for malignancies of other organs, careful follow-up is necessary due to the high risk of pancreatic cancer development. Furthermore, initial operation with minimally invasive surgery may reduce adhesion and facilitate subsequent surgeries.

**Conclusion:**

We have provided evidence that supports the importance of a careful follow-up of IPMNs, even if they are low risk.

## Highlights

• Intraductal papillary mucinous neoplasm of the pancreas is found incidentally.
• A pre-existing neoplasm led to pancreatic tail cancer in one patient.
• Due to careful follow-up of the neoplasm, the secondary cancer was detected rapidly.
• It was resected successfully by laparoscopic distal pancreatectomy.
• Careful follow is necessary if this neoplasm is detected.

## 1 Introduction

Intraductal papillary mucinous neoplasm (IPMN) is often recognized incidentally during examination of organs, other than the pancreas, for malignancy. Follow-up is recommended even for IPMNs with a low risk of malignancy, because malignant transformation may occur over time. The incidence of pancreatic cancer (over a 5-year period) in patients with IPMN is reported to be 2.5%–2.8% [1], [2], [3], [4], [5], [6], [7]. Therefore, IPMN is considered a risk factor for pancreatic cancer. We report a case of pancreatic tail cancer that developed separately from a preexisting IPMN after minimally invasive esophagectomy for cancer of the esophagogastric junction; the cancer was resected successfully by laparoscopic distal pancreatectomy.

## 2 Presentation of case

**Fig. 1:**
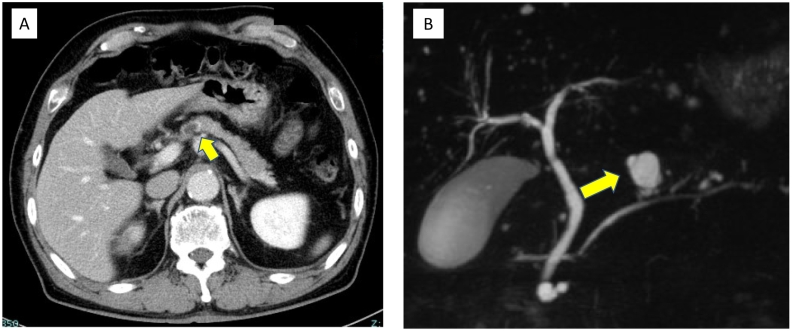
Imaging studies during the preoperative examination for esophagogastric junction cancer. A: Contrast-enhanced computed tomography. A cystic lesion is observed in the pancreatic body. B: Magnetic resonance cholangiopancreatography: cystic lesion connected to the main pancreatic duct is recognized in the pancreatic body.

**Fig. 2:**
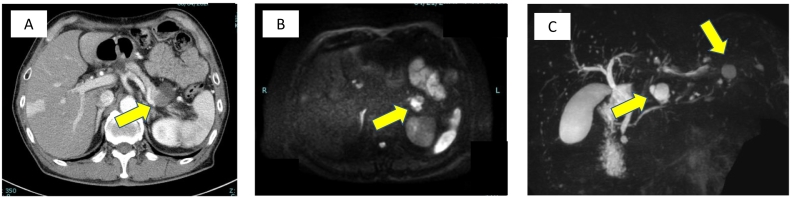
Imaging studies at the time of pancreatic tail cancer diagnosis. A: Contrast-enhanced computed tomography. A solid lesion with a cystic component in the pancreatic tail is observed. B: MRI (diffusion-emphasizing image). A high signal is noted in the pancreatic tail. C: Magnetic resonance cholangiopancreatography: Cystic lesions are observed in the pancreatic body and tail.

**Fig. 3:**
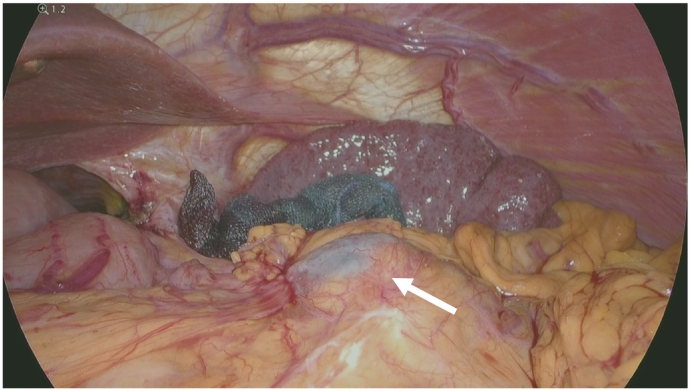
Intraoperative view. Adhesions around the pancreas appear mild. Cystic lesion is recognized in the tail of the pancreas (indicated by the arrow).

**Fig. 4:**
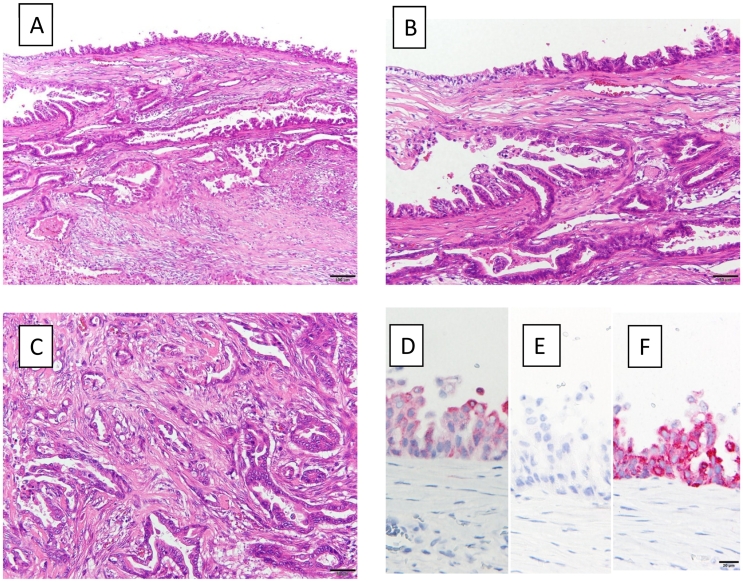
Histopathological findings of the resected specimen. A: Upper part of the lumen of the cystic lesion. The transition from the intraductal component to the invasive carcinoma is seen. HE stain, low-power view. B: Intraepithelial proliferation of neoplastic cells with high-grade dysplasia, forming papillary projections. HE stain, high-power view. C: Invasion of neoplastic cells forming tubules into the stroma. HE stain, high-power view. D: Immunohistochemistry for MUC1. Neoplastic cells show positive staining. E: Immunohistochemistry for MUC2. Neoplastic cells are not stained. F: Immunohistochemistry for MUC5AC. Neoplastic cells show strongly positive staining. HE: hematoxylin-eosin staining.

The patient was a 72-year-old man who underwent thoracoscopic lower esophagectomy and laparoscopic gastric conduit reconstruction for esophagogastric junction cancer. He had undergone surgery for ascending colon cancer 20 years ago. We were unable to ascertain any other special information relating to his medical/surgical/family/psychosocial/pharmacological history. A branch-duct IPMN was confirmed in the pancreatic body by a preoperative examination. On magnetic resonance cholangiopancreatography and endoscopic ultrasonography (EUS), the IPMN did not show any significant features such as high-risk stigmata or any other indications for resection (Fig. 1A, B); therefore, follow-up observation was recommended. Pathologically, the cancer was identified as an adenocarcinoma, and the pathological stage was pT3pN0M0 pStage II. No postoperative adjuvant chemotherapy was administered. For postoperative work-up and follow-up for the IPMN, blood tests and computed tomography (CT) were regularly performed every 3 months. The carbohydrate antigen 19–9 level increased gradually after the operation. Nine months after the operation, a lesion appeared in the tail of the pancreas. CT and MRI revealed a solid lesion with a cystic mass on the distal side (Fig. 2A–C). A biopsy of the solid lesion was performed using EUS-fine needle aspiration; histopathological examination resulted in the diagnosis of an adenocarcinoma. The lesion was considered to be a pancreatic ductal adenocarcinoma (PDAC) concomitant with IPMN that was accompanied by a retention cyst. We administered neoadjuvant chemotherapy (gemcitabine + S-1) based on the decision of a multidisciplinary conference. Subsequently, laparoscopic distal pancreatectomy was performed by YM (Fig. 3). At the time of the operation, the adhesion around the pancreas was relatively mild, even though the surgical field was close to the site of the previous operation. The operation time was 210 min, and the amount of blood loss was 300 mL. The postoperative course was uneventful, and the patient was discharged from the hospital on the 12th postoperative day. Histopathological examination of the excised mass in the tail of the pancreas revealed intraductal papillary mucinous carcinoma, and the pathological stage was ypT3ypN0yM0 ypStage IIA (Fig. 4A–C). The surgical margins were free of tumor cells. Immunohistochemically, the tumor cells were positive for MUC1 and MUC5AC, but negative for MUC2 (Fig. 4D–F). These findings were consistent with that of a pancreatobiliary type. Adjuvant chemotherapy was administered, and six months have passed since the operation without recurrence until the time of writing this manuscript. This case has been reported in line with the SCARE criteria [8].

## 3 Discussion

During follow-up of an IPMN in the pancreatic body identified during preoperative examination for a minimally invasive esophagectomy for esophagogastric junction cancer, a solid lesion with a cystic component in the pancreatic tail was recognized 9 months later. It was diagnosed as pancreatic tail cancer, and radical resection could be performed laparoscopically due to early detection.

IPMN is a pancreatic neoplasm that occurs in the pancreatic ductal epithelium and is the most frequently occurring pancreatic cystic neoplasm. It is often discovered incidentally in medical examination images. IPMN has been reported to be frequently associated with extrapancreatic malignancy (reported rates: 17%–38%) [9], [10], [11], [12], [13], [14], [15]. Although Kawakubo et al. [13] and Malleo et al. [15] prospectively tracked the occurrence of extrapancreatic malignancy in IPMN cases and concluded that the incidence was not significantly different from that in the general population, an association between extrapancreatic malignancy and IPMN is often encountered in clinical practice. Similarly, asymptomatic IPMNs are often observed in preoperative images of cancers of other organs. Yoon et al. [16] reported that 24% of the IPMN cases were diagnosed at the time of preoperative examination for cancers in other organs. Reid-Lomardo et al. reported that patients with IPMN have a higher risk of developing Barrett's esophagus (a precursor lesion of adenocarcinoma of esophagogastric junctional cancer) and esophageal cancer compared to healthy subjects [17].

Surgical resection is recommended for IPNMs with signs of malignancy, such as high-risk stigmata, according to guidelines [18]. Even for IPMN without signs of malignancy, regular surveillance is recommended, because such IPMNs may progress over time. Furthermore, PDAC may develop apart from the IPMN (PDAC concomitant with IPMN). Yamaguchi et al. reported 7 cases of PDAC concomitant with IPMN in 2002 [19]. Matsuda et al. reported that the proportion of cases with PDAC concomitant with IPMN was 10.6% among resected pancreatic cancer cases [20]. In the follow-up of IPMN cases without indications of resection, the 5-year incidence of pancreatic cancer was reported to be 2.2%–8.8% [1], [2], [3], [4], [5], [6], [7]. Therefore, IPMN is considered to be a risk factor for pancreatic cancer. Ideno et al. [4] reported that the cumulative incidence of pancreatic cancer in IPMN cases was 3.0% in 5 years and increased to 8.8% in 10 years, suggesting that the observation period should be more than 5 years. Therefore, even in malignancies of organs other than the pancreas, attention should be paid to the existence of IPMNs, and the occurrence of pancreatic cancer should be taken into consideration if an IPMN is recognized.

In recent years, the improvement of cancer treatment has increased the number of cases in which multiple surgeries are performed for cancers of different organs. Endoscopic surgery has been reported to cause fewer postoperative adhesions and decrease the difficulty of reoperation. Li et al. [21] analyzed patients who underwent common bile duct exploration after biliary tract operations, and showed that postoperative adhesions were less severe in patients who had previously undergone laparoscopic surgery as compared to in those who had previously undergone open surgery. Kinoshita et al. [22] analyzed cases of repeated hepatectomy for hepatocellular carcinoma and suggested that the introduction of laparoscopic surgery in the initial operation might reduce the difficulty of the secondary operation. In the current case, intraperitoneal adhesions were less due to the initial minimally invasive surgery for the esophagogastric junction cancer; laparoscopic surgery for the pancreatic tail cancer was found to be relatively easier. Moreover, as additional surgery may be required due to the occurrence of cancer in another organ or for remnant pancreatic cancer in the future, it would be useful to perform endoscopic surgery right from the first operation.

## 4 Conclusion

As a result of a follow-up examination of an IPMN in the pancreatic body (diagnosed at the time of minimally invasive surgery for esophagogastric junction cancer), pancreatic tail cancer was identified and laparoscopically resected in a timely manner. If IPMN is found during preoperative examination for malignancies of other organs, careful follow-up is necessary due to the high risk of pancreatic cancer development. Furthermore, initial operation with minimally invasive surgery may reduce adhesions and facilitate subsequent surgeries.

## Sources of funding

None.

## Ethical approval

Not applicable.

## Consent

Written informed consent was obtained from the patient for publication of this case report and accompanying images. A copy of the written consent is available for review by the Editor-in-Chief of this journal on request.

## Research registration

Not applicable.

## Guarantor

Masato Watanabe.

## Provenance and peer review

Not commissioned, externally peer-reviewed.

## CRediT authorship contribution statement

HK designed and drafted the manuscript. YM and MW were responsible for the revisions. SH and SN were responsible for pathological findings and interpretations. The remaining co-authors participated in discussions of manuscript content. All authors read and approved the final manuscript.

## Declaration of competing interest

None.
